# Effects of Salinity Stress on Grasspea (*Lathyrus sativus* L.) and Its Wild Relatives: Morpho-Physiological Insights at the Seedling Stage

**DOI:** 10.3390/plants14111666

**Published:** 2025-05-30

**Authors:** Khawla Aloui, Outmane Bouhlal, Hasnae Choukri, Priyanka Gupta, Keltoum El Bouhmadi, Noureddine El Haddad, Khadija El Bargui, Fouad Maalouf, Shiv Kumar

**Affiliations:** 1 International Center for Agricultural Research in the Dry Areas (ICARDA), Rabat 10112, Morocco; o.bouhlal@cgiar.org (O.B.); hasnae.choukri@um6p.ma (H.C.); k.elbargui@gmail.com (K.E.B.); 2 Laboratory of Ecology and Environment, Ben M’Sick Faculty of Sciences, University Hassan II, Casablanca 20800, Morocco; keltoum.elbouhmadi@gmail.com; 3 AgroBioSciences Program, College of Agriculture and Environmental Science, Mohammed VI Polytechnic University, Lot 660 Hay Moulay Rachid, Ben Guerir 43150, Morocco; noureddine.elhaddad@um6p.ma; 4 Laboratoire de Biotechnologie et de Physiologie Végétales, Centre de Recherche BioBio, Faculté des Sciences, Mohammed V University, Rabat 10112, Morocco; 5 Département de Phytologie, Institut de Biologie Intégrative et des Systèmes Pavillons Charles-Eugène Marchant, Université Laval, Québec, QC G1V 4G2, Canada; priyanka.gupta.1@ulaval.ca; 6 Department of Chemistry, LS3MN2E-CERNE2D, Faculty of Sciences, Mohammed V University, Rabat 10090, Morocco; 7 International Center for Agricultural Research in the Dry Areas (ICARDA), Beirut 1108 2010, Lebanon; f.maalouf@cgiar.org; 8 International Center for Agricultural Research in the Dry Areas (ICARDA), New Delhi 110012, India

**Keywords:** grasspea, salt stress, seedling stage, morpho-physiological characteristics, tolerant genotypes

## Abstract

Salinity is a critical abiotic stress influencing plant growth. However, its effect on grasspea (*Lathyrus sativus* L.) remains insufficiently explored. The present study screened 24 germplasm accessions representing 11 *Lathyrus* species at the seedling stage at 0, 100, and 150 mM NaCl concentrations using a hydroponic system. Our findings indicated that salt stress had a significant effect on all assessed traits, including a reduction in relative leaf water content and SPAD index, a decline in the length and biomass of shoots and roots, and an elevation in their corresponding dry contents. The grasspea accessions displayed a wide range of responses to salt stress. This variation allowed the identification of nine tolerant accessions at both stress levels, belonging to cultivated and wild relative species, specifically *LAT 495*, *IG 65117*, *L.OCH*, *IG 65273*, *IG 64931*, *IG 114526*, *IG 64892*, *IG 66065*, and *IG 65018*. Four accessions, namely *IG 110632*, *IG 114531*, *IG 65133*, and *IG 66026*, demonstrated tolerance only at 100 mM NaCl concentration. Through identifying these promising accessions, our research offers crucial insights for the initial screening of tolerant genotypes in grasspea, setting the stage for further studies to decipher the intricate mechanisms of salinity tolerance in these accessions.

## 1. Introduction

Agricultural productivity in arid and semi-arid regions of the world encounters substantial challenges, mainly stemming from environmental instability in these areas that imposes various abiotic stresses, hindering food security [1]. One such challenge is soil salinity, amplified by limited rainfall that fails to adequately flush salts and excess sodium ions from the rhizosphere [2]. Additionally, global practices such as irrigation, intensified crop cultivation, and the excessive application of chemical fertilizers decrease the osmotic potential of soil solution [3]. Soil salinization has extended to over 100 countries [4]. As per the FAO [5], the worldwide expanse of salt-affected soils encompasses 424 million hectares of topsoil (0–30 cm) and 833 million hectares of subsoil (30–100 cm), based on 73% of the mapped land thus far. This distribution is anticipated to escalate in future climate change scenarios, including elevated temperatures, causing increased evaporation. This trend is projected to intensify soil salinization, potentially affecting up to 50% of all arable land by 2050 [6].

The negative impacts of salt stress on plant growth, development, and metabolism arise from alterations in crucial molecular, morpho-physiological, and biochemical processes [7]. These include failed germination due to insufficient seed water absorption and Na^+^ and Cl^−^ ions accumulation [8,9], generated osmotic stress, reduced relative water content, damaged photosynthetic functions, and weakened seedling vigor [10,11]. Additionally, hyper-osmotic shock and ionic imbalance in plant cells disrupt redox homeostasis, generating oxidative stress to the cellular biomolecules due to excessive reactive oxygen species (ROS) [12,13]. These factors collectively decrease crop yield with low seed quality [7,14]. However, the severity of these effects and the tolerance mechanisms in response to salt stress differ based on the plant species, the genotype within that species, and the stage of the plant [15,16].

Being a drought-tolerant food crop, grasspea (*Lathyrus sativus* L.) is recognized for its relative tolerance to salinity [17,18,19,20]. The broad spectrum of resistance to different abiotic stresses, coupled with its high seed protein content (up to 29.9% *w*/*w*) [21,22] and efficient atmospheric nitrogen fixation (up to 124 kg/ha) [23,24], renders the crop an intriguing object for breeding programs [25,26]. Considering the crucial role of salinity-tolerant genotypes, recent investigations have concentrated on comprehending the salinity stress response in grasspea. These inquiries have revealed the detrimental impacts of increased salt concentrations on the fresh weight, seedling length, and the integrity of cellular membranes in both root and shoot tissues [27,28,29]. Moreover, the past studies also suggested that the salinity stress imposed on grasspea genotypes triggered the mechanisms for the proficient sequestration of detrimental ions [30]. Additionally, other research highlights the remarkable plasticity of the photosynthetic apparatus of grasspea, contributing to an effective mechanism for tolerating salinity stress [31].

Nevertheless, there is a need for additional insights into the impact of salinity on grasspea to sustain high crop productivity and mitigate potential yield losses. Therefore, using a hydroponic system, this investigation was designed to evaluate the morpho-physiological responses of grasspea accessions at the seedling stage to different concentrations of NaCl. The utilization of cultivated and wild relative species stems from leveraging genetic diversity from both gene pools, especially *Lathyrus* wild relatives that are vital donors of beneficial alleles to overcome various production constraints, including salinity stress [32]. By exploring and harnessing these valuable genetic resources, our study identified grasspea accessions exhibiting moderate to high salinity tolerance, serving as valuable candidates for breeding programs.

## 2. Results

### 2.1. Genotype, Treatment, and Their Interaction Effects on the Variation in Investigated Traits

**Figure 1:**
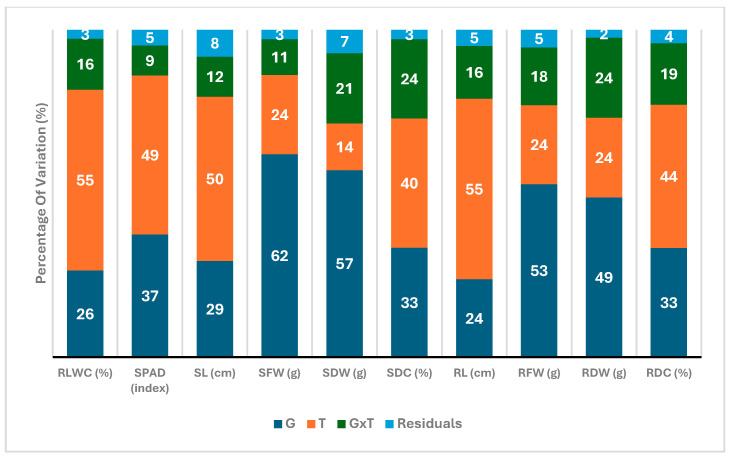
Percentage of variation explained by different sources of variation: genotype (G), treatment (T), and their interaction (G × T). RLWC, relative leaf water content; SPAD, leaf greenness index; SL, shoot length; SFW, shoot fresh weight; SDW, shoot dry weight; SDC, shoot dry content; RL, root length; RFW, root fresh weight; RDW, root dry weight; RDC, root dry content.

To evaluate the effects of genotype (G), treatment (T), and their interactions (G × T), along with calculating the broad-sense heritability for various traits, we performed a combined analysis of variance (Table 1). The effect of G, T, and G × T significantly (*p* < 0.001) affected all examined traits. Broad-sense heritability values ranged from 0.8 to 0.9, highlighting the significant effects due to the genotype component. Furthermore, the coefficient of variation (CV) pointed to a broad variability among the traits. The difference explained by each source of variation (Figure 1) revealed G and T effects as the main source of variation for all traits. For instance, traits like root fresh weight (RFW) and root dry weight (RDW) exhibited high variability (53% and 49%), with CV values of 20% and 17%, respectively, largely influenced by genotype. On the other hand, Relative Leaf Water Content (RLWC) revealed the least variability, with a CV of 3%, primarily due to treatment effects (55%). Shoot dry weight (SDW) and shoot fresh weight (SFW) depicted CV values of 13 and 12%, respectively, with genotype explaining the highest share of the variance (57% and 62%, respectively).

Moderate variability was observed in the SPAD index and Shoot Dry Content (SDC), with CV values of 7 and 5%, respectively. Both genotypes (37 and 33%) and treatment (49 and 40%) played significant roles in those traits, respectively. Similarly, shoot length (SL) and root length (RL), respectively, showed notable variability, with CV values of 12 and 10%, and treatment effects being predominant (50 and 55%). However, the G × T interaction particularly impacted RDW and RDC (24% each), and SDW and SDC (21 and 24%), while having the least influence on the SPAD index trait (9%).

### 2.2. Phenotypic Variation for Various Traits in Response to Salt Stress Treatments

**Figure 2:**
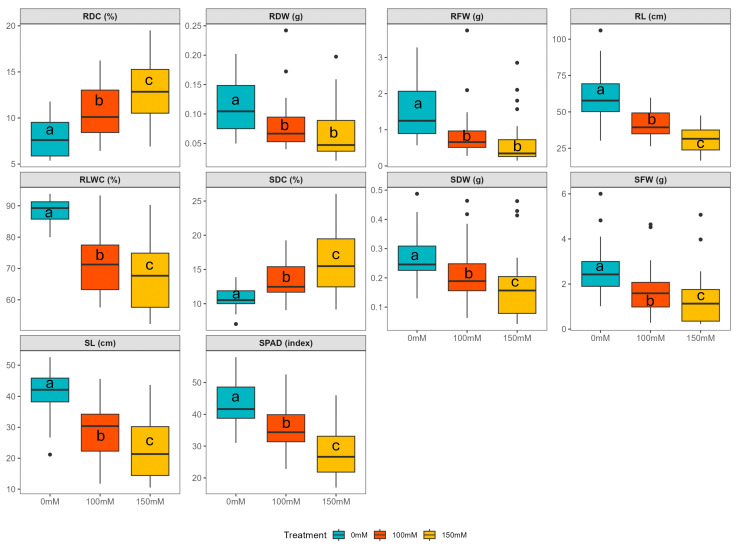
Boxplot distributions of measured traits in grasspea (*Lathyrus sativus* L.) and its wild relatives accessions subject to no stress, 100 mM NaCl, and 150 mM NaCl. Different letters (a, b, c) indicate statistically significant differences between groups at *p* < 0.05 according to Duncan’s Multiple Range Test (DMRT). RLWC, relative leaf water content; SPAD, leaf greenness index; SL, shoot length; SFW, shoot fresh weight; SDW, shoot dry weight; SDC, shoot dry content; RL, root length; RFW, root fresh weight; RDW, root dry weight; RDC, root dry content.

Wide ranges of phenotypic responses were observed across various evaluated traits, with significant differences between the treatments (*p* ≤ 0.05) (Table S1; Figure 2). RLWC and the SPAD index, reflecting water retention and chlorophyll levels, decreased by 19 and 16% under 100 mM NaCl, and further reductions of 23 and 35% were observed under 150 mM NaCl, respectively. Both SL and RL showed notable decreases, by as much as 30 and 31% under 100 mM NaCl, and 43 and 48% under 150 mM NaCl, underscoring the detrimental effects on overall plant growth. Similarly, SFW and RFW declined by 33 and 41% under 100 mM NaCl, and by 50 and 56% under 150 mM NaCl, respectively, indicating significant impacts on biomass formation. Additionally, SDW and RDW indicated decreases of 22 and 27% under 100 mM NaCl, and 37 and 36% under 150 mM NaCl, respectively. In contrast, SDC and RDC exhibited increases of 23 and 37% under 100 mM NaCl, and 47 and 67% under 150 mM NaCl, respectively.

### 2.3. Correlation Assessment of Evaluated Traits Under Control and Salt Stress Conditions

**Figure 3:**
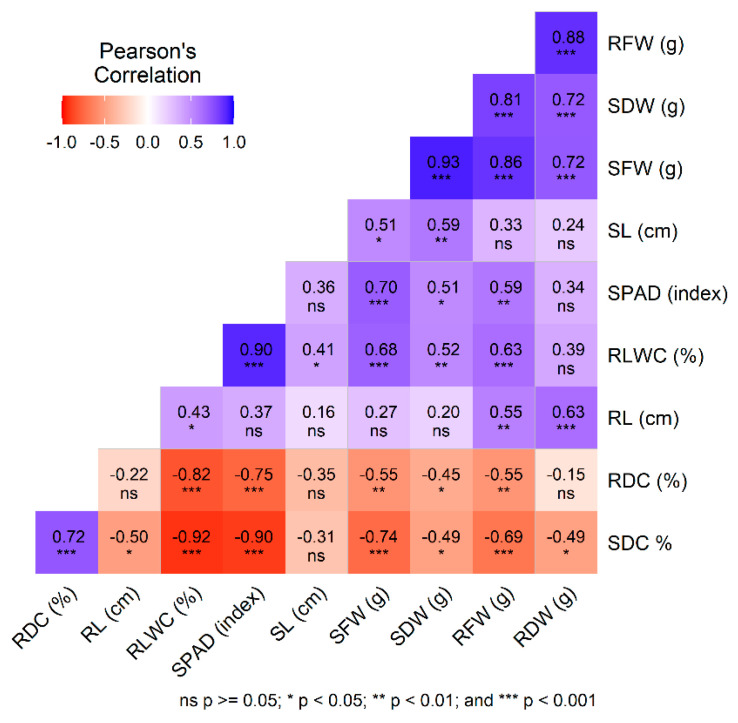
Pearson correlations between various trait combinations in grasspea (*Lathyrus sativus* L.) and its wild relatives under no stress condition. The color legend shows the correlation coefficients and the corresponding colors. * significant at *p* < 0.05, ** significant at *p* < 0.01, *** significant at *p* < 0.001.

**Figure 4:**
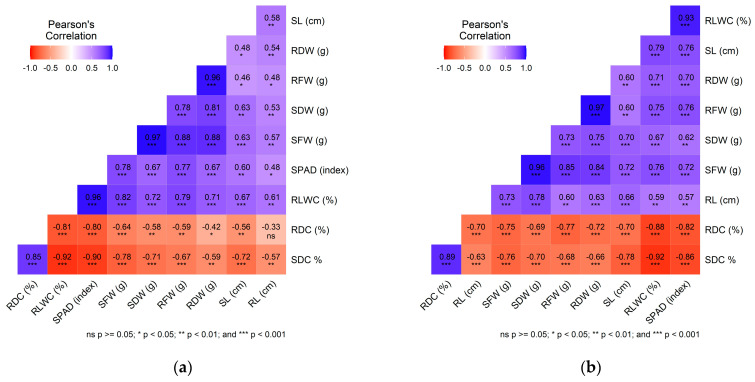
Pearson correlations between various trait combinations in grasspea (*Lathyrus sativus* L.) and its wild relatives under (**a**) 100 mM NaCl and (**b**) 150 mM NaCl. The color legend shows the correlation coefficients and the corresponding colors. * significant at *p* < 0.05, ** significant at *p* < 0.01, *** significant at *p* < 0.001.

Pearson correlation analysis was conducted to examine the relationships between various trait combinations under conditions of no stress (Figure 3) and salinity stress at 100 mM and 150 mM NaCl concentrations (Figure 4). Throughout all treatment conditions, there were consistently high positive correlations among biomass-related traits, such as SFW, SDW, RFW, and RDW. Notably, a strong negative correlation persisted between RDC and SDC against RLWC and the SPAD index, even when subjected to stress treatments. Additionally, the RLWC and the SPAD index maintained significant positive correlations with SFW and RFW across all conditions. RL consistently displayed positive correlations with RLWC and SFW and showed significant negative relationships with SDC. Similarly, SL was positively associated with SDW across all treatments.

Under stress conditions, the relationship between RL and RDC intensified negatively with higher salinity levels, becoming strongly negative at 150 mM NaCl. Likewise, the negative correlation between RL and SDC shifted from significant under no stress to moderate and then highly significant with increasing salinity levels at 100 mM and 150 mM NaCl, respectively. Moreover, the correlation between SL and SDC evolved from nonsignificant under no stress conditions to strongly positive under both stress treatments.

### 2.4. Multivariate Analysis

**Figure 5:**
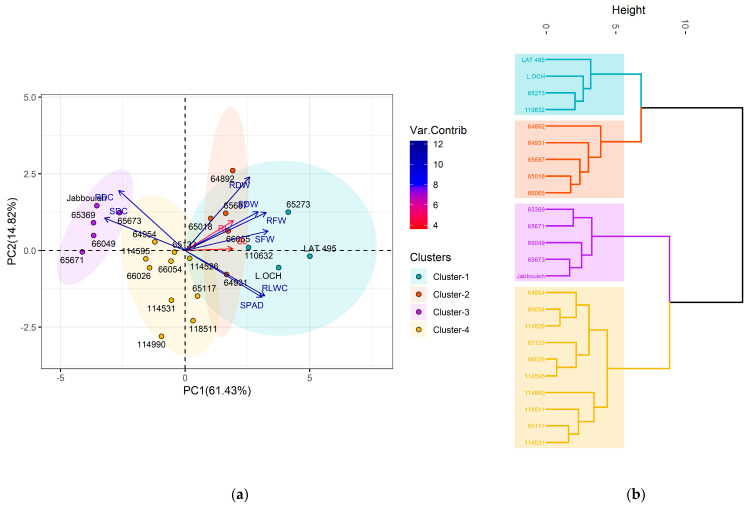
Principal component analysis (**a**) and hierarchical analysis (**b**) based on various traits contributing to the variability in grasspea (*Lathyrus sativus* L.) and its wild relatives under no stress conditions.

Principal component analysis (PCA) was conducted on the evaluated traits to simplify the visualization of variability and the spectrum of responses within the grasspea accessions to the three salt treatments (Table S2), while the PCA biplot and hierarchical cluster analysis differentiated the studied accessions into four unique groups (Table S3). Under no stress conditions (Figure 5), the first two components (PC1 and PC2) accounted for 61 and 15% of total variability, respectively. RLWC and the SPAD index showed strong positive contributions to PC1 and negative contributions to PC2, while SDC exhibited an inverse pattern, with a negative contribution to PC1 and a positive one to PC2. The largest contributors to PC1 were SFW and RFW, both positively correlated. RDW and RDC had a positive relationship with PC2, influencing its variation significantly. Based on the hierarchical cluster analysis, cluster 1, with four accessions, was notable for high RLWC, the SPAD index, SL, SFW, RFW, SDW, and RDW, presenting moderate RL and the lowest SDC and RDC. Cluster 2 consisted of five accessions with moderate to high RLWC and SL, moderate SPAD index, SFW, and SDW and exhibited low to moderate SDC, high RL, moderate RFW, high RDW, and moderate RDC. Cluster 3 contained five accessions displaying low RLWC and SPAD index, moderate SL, the lowest SFW, low SDW, high SDC, low RL, RFW, moderate RDW, and high RDC. Cluster 4 had ten accessions with moderate RLWC and SPAD index, moderate to high SL, low SFW, low to moderate SDW, moderate SDC, RL, low RFW, moderate RDW, and RDC.

**Figure 6:**
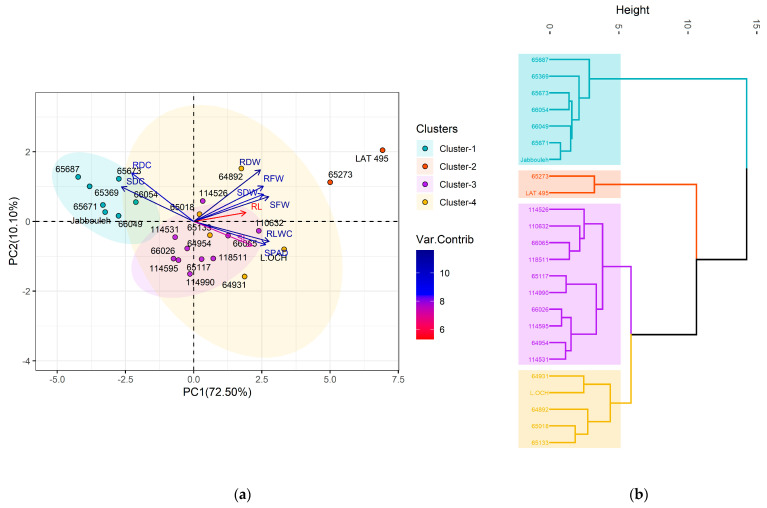
Principal component analysis (**a**) and hierarchical analysis (**b**) based on various traits contributing to the variability in grasspea (*Lathyrus sativus* L.) and its wild relatives under 100 Mm NaCl.

**Figure 7:**
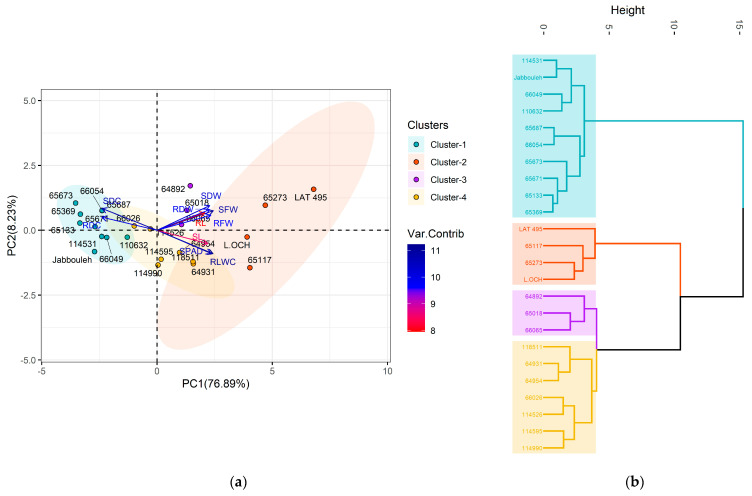
Principal component analysis (**a**) and hierarchical analysis (**b**) based on various traits contributing to the variability in grasspea (*Lathyrus sativus* L.) and its wild relatives under 150 Mm NaCl.

Under stress treatments, the variability explained by PC1 and PC2 accounted for 73 and 10%, respectively, under 100 mM NaCl treatment (Figure 6), whereas at 150 mM NaCl (Figure 7), they explained 77 and 8%. Under both stress conditions, the variation in PC1 was significantly and positively influenced by RLWC, SFW, and the SPAD index, and negatively correlated with SDC. For PC2, at 100 mM NaCl condition, RDW, RDC, SFW, and SDC were the main traits contributing positively to its variation. When hierarchically clustering the accessions (Table S4), cluster 1 expanded to seven accessions, characterized by low RLWC and SPAD index, moderate SL, low SFW, SDW, high SDC, moderate RL, RFW, low RDW, and high RDC. Cluster 2, with two accessions, showed high RLWC, SPAD index, SL, SFW, SDW, low SDC, high RL, RFW, RDW, and low RDC. Clusters 3 and 4, with ten and five accessions, respectively, maintained moderate RLWC and SPAD index, high SL, and moderate SFW, SDW, SDC, RFW, with varying degrees of RL (moderate in group 3 and high in group 4), RDW, and RDC (low to moderate in group 3 and moderate in group 4).

Meanwhile, at the 150 mM NaCl level, RLWC and the SPAD index exerted a negative influence on PC1, whereas SDW, SDC, and RDW positively and significantly influenced the variation in PC2. Under those conditions, cluster 1 included ten accessions with low RLWC, SPAD index, SL, SFW, SDW, high SDC, moderate RL, low RFW, RDW, and high RDC. Cluster 2, with four accessions, presented high levels across RLWC, SPAD index, SL, SFW, SDW, low SDC, RL, RFW, RDW, and low RDC. Cluster 3 had three accessions with moderate RLWC, SPAD index, varying SL, moderate SFW, high SDW, SDC, high RL, RFW, RDW, and moderate RDC. Lastly, cluster 4 consisted of seven accessions with moderate RLWC, SPAD index, varying SL, moderate SFW, SDW, SDC, high RL, low RFW, RDW, and moderate RDC.

### 2.5. Salinity Tolerance

**Figure 8:**
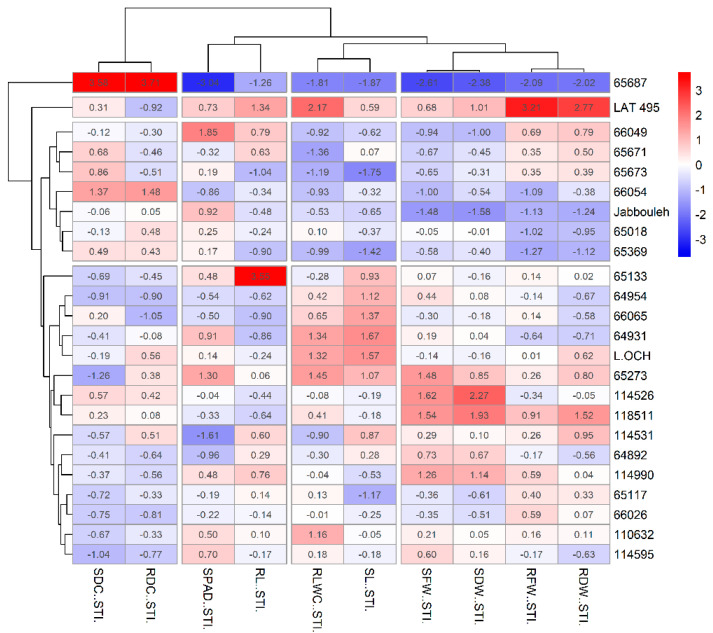
Heatmap and hierarchical clustering for evaluated traits under 100 Mm NaCl in grasspea (*Lathyrus sativus* L.) and its wild relatives. Accession names on the vertical axis. Trait indices named on the horizontal axis. Bright blue color indicates the lowest values, while bright red color indicates the highest values for each trait.

**Figure 9:**
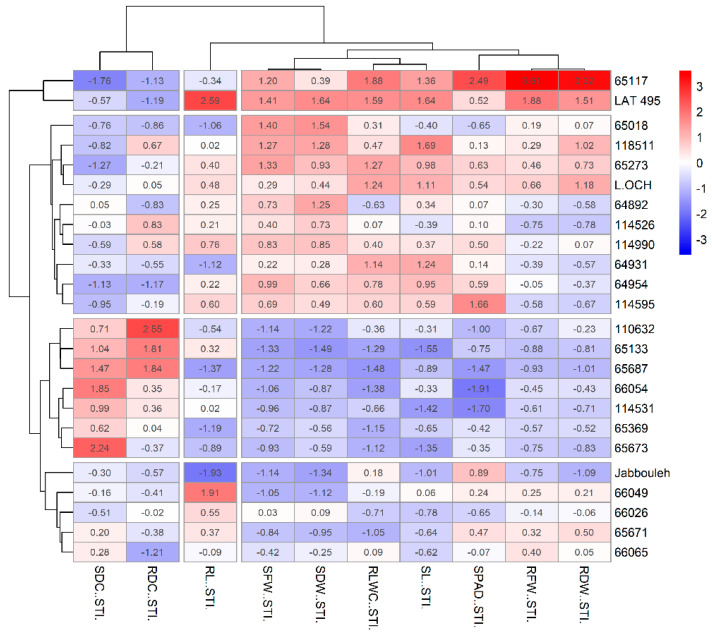
Heatmap and hierarchical clustering for evaluated traits under 150 Mm NaCl in grasspea (*Lathyrus sativus* L.) and its wild relatives. Accession names on the vertical axis. Trait indices named on the horizontal axis. Bright blue color indicates the lowest values, while bright red color indicates the highest values for each trait.

The results of the hierarchical cluster analysis were supported by a heatmap–cluster analysis (Figure 8 and Figure 9) based on the Salt Tolerance Index (STI) across two salinity stress levels. At a concentration of 100 mM NaCl, the heatmap organized traits into correlated groups; it paired SDW and SFW along with their corresponding RDW and RFW, as one cluster, aligned SL and RLWC in another, associated RL with SPAD, and linked RDC with SDC in separate groups, leading to the categorization of accessions into four distinct clusters.

The first cluster included the most susceptible accession *IG 65687*, notable for its minimal biomass and physiological indices but highest in RDC and SDC indices. In contrast, the second cluster contained the most tolerant accession *LAT 495*, marked by the highest RDW, RFW, and RLWC tolerance indices but lower RDC and moderate SDC indices. Reduced SDW, SFW, SL, and RLWC, but higher SDC indices, characterized the third cluster, containing seven susceptible accessions, including the check *Jabbouleh*. The fourth cluster assembled accessions with moderate tolerance, displaying moderate to high biomass and physiological traits, coupled with low to moderate RDC and SDC levels. Notably, accession *IG 65133* demonstrated the highest RL index within this cluster.

At 150 mM NaCl concentration, the heatmap unveiled three trait groups: one consisting of biomass and physiological traits, another for root length alone, and a third for RDC and SDC together. Two highly salt-tolerant accessions were clustered, displaying the most advantageous combination of biomass and physiological indices. In this cluster, *LAT 495* presented the highest RL index, while *IG 65117* recorded the lowest SDC index. The second cluster grouped ten moderately tolerant accessions, characterized by high SDW and SFW and generally moderate to high levels of other trait indices, alongside a low SDC index. The third cluster included highly susceptible accessions with the lowest overall biomass and physiological indices, the lowest RL index, and elevated RDC and SDC indices. Lastly, the fourth cluster, with five accessions including the check *Jabbouleh*, represented moderately susceptible accessions, marked by low SL, RLWC, SDW, and SFW.

## 3. Discussion

Soil salinity stands as a critical challenge that hampers the growth and yield of legume crops [2]. In this scenario, identifying genotypes that can tolerate salinity becomes a crucial measure for ensuring the ongoing and sustainable production of legume crops [33]. Hence, in this research, 24 germplasm accessions representing 11 *Lathyrus* species, previously assessed for their resilience to heat and drought stress [34], were examined to determine their morpho-physiological reactions to three varying NaCl concentration treatments.

Our findings demonstrated that salt stress at the seedling stage had a notable effect on grasspea (*Lathyrus sativus* L.) and its wild relatives accessions; this impact was more intensified with rising concentrations. Consistent with previous research on grasspea [30,31,35,36,37], the physiology of the seedlings was adversely affected by the stresses applied, indicating that salt stress can affect the physiological processes of the plants either directly or indirectly [38,39]. In this investigation, salt stress led to a reduction in relative leaf water content by 19% at 100 mM and 23% at 150 mM, highlighting its value as a key indicator of stress severity and one of the most dependable markers of growth and biochemical responses [40]. This decline is likely attributable to disrupted water relations within the plant [41]. The buildup of salt in the hydroponic solution results in a higher solute concentration within the root zone, leading to a reduction in both water potential and osmotic potential, thereby impairing the ability of plants to uptake water [42]. In addition, the SPAD index, which has a positive correlation with chlorophyll levels, exhibited a decline as stress intensity increased. It showed a 16% decrease at 100 mM and a 35% decrease at 150 mM, reflecting the adverse effects of saline conditions on the photosynthetic machinery. This trend was also observed in certain reported grasspea genotypes and other legume crops [30,31,35,43,44,45,46], stemming from a disruption in chlorophyll synthesis and an increase in pigment degradation [47]. This disruption is largely due to the negative impact of Na^+^ ions on the activity of 5-aminolevulinic acid (ALA) dehydrogenase, an enzyme crucial for the production of ALA, a precursor for porphyrins and subsequently chlorophylls [48,49]. Furthermore, salt stress may alter the expression of genes involved in porphyrin biosynthesis and increase the activity of chlorophyllase, leading to accelerated chlorophyll degradation [50].

Physiological alterations due to salt stress significantly affected plant growth, typically assessed through shoot and root dimensions, resulting in shortened lengths and reduced fresh weights of both components when compared with no-stressed plants. In contrast with the findings from other studies [29,51,52], our results reveal more pronounced inhibition of root growth compared with shoot growth. Specifically, shoot length decreased by 30% at 100 mM and by 43% at 150 mM, while root length experienced even greater reductions of 31% at 100 mM and 48% at 150 mM. Indeed, questions remain regarding the more susceptible part of the plant (shoots or roots) to salinity [53]. In line with our observations, Barbara et al. [27] have pointed out that the roots of grasspea exhibit greater sensitivity to salinity compared with the shoots. This may stem from the harmful effects of salt stress, particularly reduced turgor pressure in radicle cells; salt stress is first recognized by the root system, where it induces osmotic stress by limiting water absorption, thereby restricting root growth and functionality prior to influencing shoot development [54]. Additionally, the accumulation of toxic ions like Na^+^ and Cl^−^ in the root zone can cause direct damage to root cells and tissues, hinder nutrient absorption, and interfere with metabolic activities crucial for root growth [55,56]. To mitigate the effects of salinity, plants may adapt morphologically and anatomically by shortening root length or volume to reduce exposure to detrimental ions, while also modifying shoot structure to enhance light absorption and gas exchange in stressful conditions [57].

In our study, the positive correlation between relative leaf water content and SPAD index with the weights of roots and shoots highlights the significant physiological challenges posed by salinity, leading to decreases in both dry and fresh plant biomass. These outcomes align with findings from prior research on grasspea [27,28,29,30,31], underscoring the widespread impacts of salinity stress on plant growth. Despite a reduction in dry weight due to salinity treatments, a negative correlation was observed with dry weight content, which serves as a crucial metric for assessing the effects of salinity stress on plants, stemming from intricate physiological processes primarily based on carbohydrates [30,58]. Even though an increase in dry matter content is commonly considered as a characteristic observed in plants that exhibit tolerance to water and chemical stress [27,59], our findings indicate an increase in dry content across both shoots and roots of grasspea accessions, with SDC and RDC showing rises of 23% and 37% under 100 mM NaCl, and 47% and 67% under 150 mM NaCl, respectively. These increases were notably more pronounced in susceptible accessions than in those that are tolerant. This increase is likely due to rapid water loss, which consequently raises their relative dry weight content. Conversely, salt-tolerant plants exhibited lower dry weight content, aligning with the observed negative correlation with relative leaf water content. This suggests that these plants are likely better at conserving water and adjusting osmotically, possibly through the synthesis of osmoregulatory and osmoprotective compounds, rather than focusing on biomass growth when faced with salinity stress [30,60]. Such a survival strategy enables them to preserve essential physiological functions and thrive in salt-affected conditions.

The 24 *Lathyrus* accessions exhibited diverse responses at the seedling stage to different salt treatments, as revealed by variance analysis, which highlighted a highly significant influence of genotype and its interaction with treatment across all measured traits. To distinguish between the evaluated *Lathyrus* accessions, PCA was employed alongside hierarchical cluster analysis. The identified clusters showed concordance with the patterns observed in a heatmap analysis based on salinity tolerance indices, identifying promising accessions with different levels of salinity stress tolerance.

Among these identified accessions, *L. sativus* accession “*LAT 495*” stood out as the most tolerant at both levels of stress, displaying high relative leaf water content and an increase in root dry biomass under salt stress. Meanwhile, *L. sativus* accession *IG 65117* demonstrated a notable adaptability to salt stress, with only a minor decline under 100 mM NaCl treatment, followed by a recovery of its physiological parameters to near-normal levels, in addition to an increase in root dry biomass. The enhanced root weight in these accessions underlines the critical role of roots in salt stress tolerance and illustrates the varied adaptive responses within *Lathyrus* accessions to escalating salinity stress. Interestingly, both accessions belong to cultivated species, which provide valuable insights in breeding efforts aimed at developing salt-tolerant grasspea varieties, while emphasizing the importance of screening cultivated species alongside wild relatives, as valuable traits may be present in both gene pools. Other accessions, including *L.OCH (Lathyrus ochrus* L.), *IG 65273 (Lathyrus annuus* L.), and *IG 64931* (*Lathyrus sativus* L.), were identified as highly tolerant, maintaining stable physiological parameters and biomass, with low dry contents in roots and shoots, indicating a strategic reallocation of resources towards sustaining growth and physiological functions over biomass accumulation in response to stress. This approach results in a lower dry weight content yet preserves essential functions and tolerance to stress. The moderate level of salinity tolerance was represented by a group of accessions including *IG 114526* (*Lathyrus sativus* L.), *IG 64892* (*Lathyrus sativus* L.), *IG 65018* (*Lathyrus inconspicuus* L.), and *IG 66065* (*Lathyrus gorgoni* Parl), showing high shoot biomass under salt stress.

On the other hand, the 150 mM NaCl concentration emerges as a critical threshold that delineates the salinity tolerance capacities of certain grasspea accessions, including *IG 110632* (*Lathyrus sativus* L.), *IG 114531* (*Lathyrus sativus* L.), *IG 65133* (*Lathyrus sativus* L.), and *IG 66026* (*Lathyrus tingitanus* L.). While these accessions demonstrated a high level of tolerance at 100 mM NaCl, their susceptibility at 150 mM NaCl concentration suggests a gradient of tolerance, pointing to the existence of varying physiological and genetic mechanisms that confer salinity tolerance.

Among the salt-tolerant accessions that were identified, four of them—*IG 65273*, *IG 64931*, *IG 65018*, and *IG 66026*—had previously been recognized for their optimal combination of low ODAP levels along with high yield and protein content when subjected to heat and drought stress conditions [34].

## 4. Materials and Methods

### 4.1. Plant Material

The experimental material consisted of 24 germplasm accessions, representing 11 *Lathyrus* species. These accessions were selected from a diversity panel of 435 germplasm sourced from the ICARDA genebank, using ODAP content, grain yield, and biomass as selection criteria (Table S4).

### 4.2. Growth Conditions and Stress Treatments

This study employed a randomized complete block design with two replications, conducted within the growth chamber of the physiology laboratory at ICARDA-Rabat, Morocco. To ensure the reliability of our screening, the experiment was repeated independently in February and March 2023 in the growth chamber of the Physiology Laboratory at ICARDA-Rabat, Morocco. Since no significant variation was found between the two runs (*p* > 0.05), data were pooled for analysis.

For each accession, uniform pre-germinated seeds were transferred to hydroponic containers filled with a modified Hoagland nutrient solution, based on the standard formulation but with adjusted concentrations of both macro- and micronutrients to better meet the nutritional requirements of legume species [61,62,63]. Salinity stress was applied after 10 days of germination. This involved subjecting the seedlings to three distinct treatments: (i) no stress with 0 mM NaCl; (ii) 100 mM NaCl; and (iii) 150 mM NaCl. The seedlings were kept under these conditions for 20 days, maintaining average day/night temperatures of 25/16 °C, relative humidities of 80/60%, and a photoperiod of 16/8 h. The following morpho-physiological traits were determined.

### 4.3. Relative Leaf Water Content

To evaluate the relative leaf water status, the method of Barrs and Weatherley [64] was employed. Fresh leaflet samples, comprising 4–5 leaflets from the uppermost branch, were gathered from accessions exposed to 0, 100, and 150 mM NaCl. The initial fresh weight of the leaflet samples was recorded before immersing them in distilled water overnight. After extracting the leaves from the water and allowing them to airdry on blotting paper, they were re-weighed to establish the turgid weight. Subsequently, the leaf samples were dried in an oven (Binder, model ED 23, Tuttlingen, Germany) at 80 °C for 24 h and re-weighed to determine the dry weight. The relative leaf water content (RLWC) was then calculated as follows:

(1) $$RLWC\left(\%\right)=\frac{FW-DW}{TW-DW}\times 100$$

where FW is the fresh weight (g), TW is the turgid weight (g), and DW is the dry weight (g).

### 4.4. Total Chlorophyll

Chlorophyll content was assessed utilizing the SPAD-502 chlorophyll meter (Minolta, Osaka, Japan), a compact, diagnostic, nondestructive, and portable device [65]. The SPAD-502 chlorophyll meter provides a nondestructive estimate of leaf chlorophyll content. Its readings have been shown to correlate positively and significantly with chlorophyll concentrations determined through destructive laboratory analyses in a range of crop species [66], demonstrating its reliability for in vivo chlorophyll assessment. In our investigation, SPAD values were determined for each plant by recording the average of three measurements from distinct leaflet samples obtained from the uppermost branches.

### 4.5. Biometric Traits

Biometric parameters were recorded at the end of the experiment, specifically 20 days after the initiation of the salt treatment. Measurements were recorded on shoot and roots separately. The lengths of both shoots and roots of the seedlings were measured, and their fresh weights were determined. For root fresh weight, excess surface water was allowed to drain naturally by gently holding the roots. Blotting was then carried out multiple times using fresh layers of laboratory tissue paper (Whatman Grade 1), applied gently until no visible water droplets remained. The absence of moisture transfer to a clean blotting paper was used as a visual indicator of dryness. Root fresh weight was then measured immediately using a calibrated analytical balance (OHAUS, Parsippany, NJ, USA). This procedure was adapted from established protocols aimed at minimizing weighing errors due to surface water retention [67,68].

Subsequently, the plant materials were oven-dried at 100 °C for 48 h in an oven (Binder, model ED 23, Tuttlinge, Germany) and re-weighed to ascertain the dry weight of both shoots and roots. This information was then used to calculate the percentage of organ dry weight content, following the provided formula:

(2) $$\%DC=\frac{Organ\;DW}{Organ\;FW}\times 100$$

where % DC represents the percentage of dry matter content, with DW denoting the dry weight (g) and FW representing the fresh weight (g).

### 4.6. Statistical Analysis

Linear mixed models implemented in *lmer* from the *lme4* R package version 1.1-35.5 were used to calculate BLUEs and BLUPs (Tables S5 and S6) and estimate the variance components [69]. The replication in each treatment was considered as a random effect for both the first and second stage analyses. In the first stage analysis, the salinity stress treatment was used as a fixed effect, while the genotypes were considered as random effects. A diagonal structure of variance across treatments was used to compute the genotypic variance and heritability in each salt treatment. The second stage analysis was performed using both genotypes and G × T as random effects. The best linear unbiased estimations (BLUEs) of all traits in each treatment were computed and compared, and significant differences were determined using the Duncan Multiple Range Test (DMRT), with a significance level set at *p* < 0.05. The BLUEs of all traits in each treatment were used to perform Pearson correlations, principal component analysis (PCA), hierarchical cluster analysis (HCA), and the Salt Tolerance Index (STI).

The Salt Tolerance Index (STI) (Tables S7 and S8) was calculated as the ratio of a trait value in plants treated with NaCl to the same trait value in control plants [70]. Heatmap hierarchical clustering was used to identify genotype clusters based on their reaction to salt stress at 100 mM and 150 mM. For each analysis, genotypes were clustered using Euclidean distance, while salt tolerance indices were clustered using the Pearson correlation coefficient.

All graphs were generated using R software version 4.1.3 and R Studio version 1.3.31093. The *METAN* Package version 6.0 was used to compute the phenotypic Pearson correlation coefficients among traits [71]. The boxplots were performed with *ggplot2* package version 2.1 [72]. The PCA was generated using *factoextra* Version 1.0.7 [73], and *FactoMineR* version 2.1 [74] packages in R, while HCA was performed using Ward’s squared Euclidean distance method with the *dendextend* R package version 1.12.0 [75]. Heatmap clustering was performed using *pheatmap* Package version 1 [76].

## 5. Conclusions

This study evaluated the responses of various grasspea accessions to salinity stress, revealing significant genotypic variation in both morphological and physiological traits under saline conditions. Growth reductions, particularly in shoot and root biomass, highlighted the detrimental effects of salt stress; however, some accessions demonstrated notable tolerance. Principal component analysis (PCA) was used in combination with hierarchical cluster analysis, and the resulting clusters aligned with the patterns revealed by the heatmap analysis of salinity tolerance indices. Consequently, LAT 495 and IG 65117 were identified as the most tolerant genotypes. Among the salt tolerance indicators, shoot dry weight (SDW), root dry weight (RDW), relative leaf water content (RLWC), the SPAD leaf greenness index, root dry content (RDC), and shoot dry content (SDC) emerged as the most reliable indicators of salinity tolerance, displaying strong correlations with overall plant performance. These traits can serve as effective selection criteria in breeding programs aimed at enhancing salt tolerance. Overall, the findings provide a valuable foundation for developing resilient grasspea cultivars adapted to saline environments, supported by effective screening methods such as PCA, cluster analysis, and heatmap visualization.

## Figures and Tables

**Table 1:** Combined analysis of variance (ANOVA), coefficient of variation, and broad-sense heritability for evaluated traits in grasspea (*Lathyrus sativus* L.) and its wild relatives.

Trait	G	T	G × T	Residual	CV (%)	H_2_
RLWC (%)	54.851 *	114.628 *	32.169 *	5.663	3.128	0.83
SPAD (index)	43.846 *	56.867 *	10.703 *	5.627	6.634	0.92
SL (cm)	46.732 *	80.04 *	19.416 *	13.058	11.633	0.86
SFW (g)	1.164 *	0.455 *	0.204 *	0.054	12.116	0.94
SDW (g)	0.008 *	0.002 *	0.003 *	0.001	12.974	0.90
SDC (%)	5.329 *	6.305 *	3.857 *	0.459	5.086	0.80
RL (cm)	96.248 *	223.002 *	65.327 *	19.826	9.912	0.80
RFW (g)	0.414 *	0.189 *	0.138 *	0.042	19.929	0.89
RDW (g)	0.002 *	0.001 *	0.001 *	0.0001	17.325	0.88
RDC (%)	5.096 *	6.691 *	2.863 *	0.624	7.519	0.84

* indicates significance at 0.001 probability level. RLWC, relative leaf water content; SPAD, leaf greenness index; SL, shoot length; SFW, shoot fresh weight; SDW, shoot dry weight; SDC, shoot dry content; RL, root length; RFW, root fresh weight; RDW, root dry weight; RDC, root dry content.

## Data Availability

Datasets generated and/or analyzed during the current study are available from the corresponding authors upon reasonable request.

## Supplementary Materials

The following supporting information can be downloaded at https://www.mdpi.com/article/10.3390/plants14111666/s1, Table S1: Descriptive statistics for evaluated traits in grasspea under no stress, 100 mM NaCl, and 150 mM NaCl., Table S2: Percentage of contribution of different traits in grasspea to major principal components with percentage variation under no stress, 100 mM NaCl, and 150 mM NaCl., Table S3: Cluster mean ± SD of evaluated traits under no stress, 100 mM NaCl, and 150 mM NaCl, Table S4: Description of the 24 accessions of 11 Lathyrus species used in the study, Table S5: Best linear unbiased estimates (BLUEs) of the evaluated traits in grasspea under no stress conditions, 100 mM NaCl, and 150 mM NaCl treatments, Table S6: Best linear unbiased predictions (BLUPs) of the evaluated traits in grasspea under no stress conditions, 100 mM NaCl, and 150 mM NaCl treatments, Table S7: Salt Tolerance Index (STI) for evaluated traits in grasspea under 100 mM NaCl salinity stress, Table S8: Salt Tolerance Index (STI) for evaluated traits in grasspea under 150 mM NaCl salinity stress.

## Abbreviations

The following abbreviations are used in this manuscript:

ALA	5-Aminolevulinic Acid
BLUPs	Best Linear Unbiased Predictions
BLUEs	Best Linear Unbiased Estimators
Cl^−^	Chloride Ion
CV	Coefficient of Variation
DC	Dry Content
DMRT	Duncan Multiple Range Test
FAO	Food and Agriculture Organization
FW	Fresh Weight (g)
G	Genotype
G × T	Genotype × Treatment Interaction
HCA	Hierarchical Cluster Analysis
ICARDA	International Center for Agricultural Research in the Dry Areas
Na^+^	Sodium Ion
NaCl	Sodium Chloride
ODAP	3-N-Oxalyl-L-α,β-diaminopropionic acid
PCA	Principal Component Analysis
PC1	Principal Component 1
PC2	Principal Component 2
RDC	Root Dry Content
RDW	Root Dry Weight
RL	Root Length
RLWC	Relative Leaf Water Content
ROS	Reactive Oxygen Species
RFW	Root Fresh Weight
SDW	Shoot Dry Weight
SDC	Shoot Dry Content
SFW	Shoot Fresh Weight
SL	Shoot Length
SPAD	Soil Plant Analysis Development (index)
STI	Salt Tolerance Index
T	Treatment
TW	Turgid Weight
